# Women decision-making capacity and intimate partner violence among women in sub-Saharan Africa

**DOI:** 10.1186/s13690-018-0253-9

**Published:** 2018-01-29

**Authors:** Bright Opoku Ahinkorah, Kwamena Sekyi Dickson, Abdul-Aziz Seidu

**Affiliations:** 1 Department of Health, Physical Education and Recreation, University of Cape Coast, Cape Coast, Ghana; 2 Department of Population and Health, University of Cape Coast, Cape Coast, Ghana

**Keywords:** Women, Decision-making capacity, Intimate partner violence, Sub-Saharan Africa

## Abstract

**Background:**

Violence against women is a common form of human rights violation, and intimate partner violence (IPV) appears to be the most significant component of violence. The aim of this study was to examine the association between women decision-making capacity and IPV among Women in Sub-Saharan Africa. The study also looked at how socio-demographic factors also influence IPV among Women in Sub-Saharan Africa.

**Methods:**

The study made use of pooled data from most recent Demographic and Health Survey (DHS) conducted from January 1, 2010, and December 3, 2016, in 18 countries in Sub-Saharan Africa. For the purpose of the study, only women aged 15–49 were used (*N* = 84,486). Univariate and multivariate logistic regression models were used to investigate the relationship between the explanatory variables and the outcome variable.

**Results:**

The odds of reporting ever experienced IPV was higher among women with decision-making capacity [AOR = 1.35; CI = 1.35–1.48]. The likelihood of experiencing IPV was low among young women. Women who belong to other religious groups and Christians were more likely to experience IPV compared to those who were Muslims [AOR = 1.73; CI = 1.65–1.82] and [AOR = 1.87; CI = 1.72–2.02] respectively. Women who have partners with no education [AOR = 1.11; CI = 1.03–1.20], those whose partners had primary education [AOR = 1.34; CI = 1.25–1.44] and those whose partners had secondary education [AOR = 1.22; CI = 1.15–1.30] were more likely to IPV compared to those whose partners had higher education. The odds of experiencing IPV were high among women who were employed compared to those who were unemployed [AOR = 1.33; CI = 1.28–1.37]. The likelihood of the occurrence of IPV was also high among women who were cohabiting compared to those who were married [AOR = 1.16; CI = 1.10–1.21]. Women with no education [AOR = 1.37; CI = 1.24–1.51], those with primary education [AOR = 1.65; CI = 1.50–1.82] and those with secondary education [AOR = 1.50; CI = 1.37–1.64] were more likely to experience IPV compared to those with higher education. Finally, women with poorest wealth status [AOR = 1.28; CI = 1.20–1.37], those with poorer wealth status [AOR = 1.24; CI = 1.17–1.32], those with middle wealth status [AOR = 1.27; CI = 1.20–1.34] and those with richer wealth status [AOR = 1.11; CI = 1.06–1.17] were more likely to IPV compared to women with richest wealth status.

**Conclusion:**

Though related socio-demographic characteristics and women decision-making capacity provided an explanation of IPV among women in sub-Saharan Africa, there were differences in relation to how each socio-demographic variable predisposed women to IPV in Sub-Saharan Africa.

## Background

Violence against women is a common form of human rights violation, and intimate partner violence (IPV) appears to be the most significant component of violence [1, 2]. IPV is associated with a wide range of negative consequences for women who are abused, including loss of pregnancy and contraction of sexually transmitted infections [3]. IPV is common in all societies, but the level and rate to which it is considered acceptable differ from one country to another [4]. All over the world, Sub-Saharan African countries have recorded the highest levels of violence against women [1]. Intimate partner violence (IPV) is widespread throughout much of sub-Saharan Africa, with an overall prevalence of 36% exceeding the global average of 30% [5]. More women in Africa are subject to lifetime partner violence (45.6%) and sexual assault (11.9%) than women anywhere in the world [5].

IPV has adverse outcomes for women, ranging from poor psychological health to adverse reproductive health effects such as poor birth outcomes [6]. In Sub-Saharan Africa, women show several psychological disorders in response to intimate partner abuse although both men and women manifest psychological symptoms in the aftermath of a physical altercation [7]. Previous studies by Pallitto et al. [8] and Salazar and San Sebastian [9] have identified strong associations between IPV and negative pregnancy outcomes. Women who reported ever experiencing physical or sexual violence by spouses have been identified to have higher odds of unintended or unwanted pregnancies. Lifetime experience of IPV has also been associated with higher odds of having a non-live birth, thus, having a pregnancy ending in a miscarriage, induced abortion, or termination [10, 11]. Moreover, reproductive health can be compromised in several ways, from raising the risk of STIs to threatening a pregnancy [12]. IPV is also associated with sexually transmitted infections [13] which in Africa carry significance as potential gateways to HIV infection [14]. Some of the risk factors for IPV in Africa mirror those found in other regions of the world such as excessive drinking or a past history of child abuse, or socioeconomic conditions such as unemployment. In addition, long-standing patriarchal traditions play a role.

African cultural beliefs and traditions promote men’s hierarchical role in sexual relationships and especially marriage [15]. Norms surrounding violence in families also change more slowly in rural areas [16]. In sub-Saharan Africa, gender relations incorporating violence reflect the legacy of conflict and hegemony which marked the colonial period [17]. According to Boonzaier [18], economic and political transformation has actually left many men without a clear position, provoking tension in the relationships between men and women. Beliefs relating to gender roles in marriage lay the groundwork for IPV in many regions of Africa. Patriarchal beliefs are not the only explanation for partner abuse but such attitudes sustain community tolerance of IPV reducing the chance for a systemic social response [19]. In sub-Saharan Africa, a significant proportion of both men and women endorse a man’s prerogative to physically discipline his wife [20] with more women than men endorsing what they view as justified abuse, such as when a wife appears to neglect the children or argues with her husband [21].

To understand the origins of intimate partner abuse in Africa, it is important to interpret the problem against the context of family life and gender roles [17, 22] The belief that women’s sexual response must be suppressed, that she should be traded for marriage by her father, and that her husband is free to take multiple wives, sets the stage for the commodification of women and the acceptance of violence in support of a husband’s effort to control [19]. In this regard, one of the key issues that seem to offer a solution to IPV is women decision making capacity, which is sometimes linked with empowerment. Empowerment is a process that gives power to the individuals who are not empowered and increases their ability to make strategic choices [23]. In line with this, women empowerment has been defined as the growth in the ability of people to make planned life choices within a context where this ability was previously denied to them [24].

Evidence suggests that, at times, women’s economic or social empowerment may undermine IPV, and at times may trigger it [25, 26]. Jewkes et al. [25] theorize that as a woman begins to become empowered she may question rigid gender roles but her empowerment may not be sufficient to insulate her from the effects of such change. According to Rahman, Hoque and Makinoda [27], the likelihood of all forms of IPV increases with participation in household decision-making. Naved and Persson [28] report similar findings. These findings suggest that women’s empowerment may influence their risk of IPV in complex and contradictory ways and that their risk may vary with specific forms of empowerment. On the contrary, a recent study in Bangladesh identified that women empowerment is becoming protective against IPV even though IPV rates remain high [29]. The absence of uniformity in the understanding of whether women empowerment results in or reduces IPV has not been explored among women in sub-Saharan Africa.

In the context of Sub-Saharan Africa, few studies have been conducted. One of such studies was conducted by Naved and Persson [30] where they found that women’s empowerment has a strong influence on their use of reproductive and maternal health service. A recent study by Cools and Kotsadam [4] also looked at resources and IPV in Sub-Saharan Africa and found that resource inequality, both within the household and at the aggregate level, is associated with more abuse. The paucity of literature that has looked at the relationship between women empowerment, especially in relation to women decision-making capacity and IPV in Sub-Saharan Africa, together with the inconsistency in findings of previous studies that have looked at women empowerment and IPV in other countries, has necessitated the need for this study. With women decision-making capacity, constituting a key component of women empowerment, the aim of this study is to examine the association between women decision-making capacity and IPV among Women in Sub-Saharan Africa. The study also looks at how socio-demographic factors also influence IPV among Women in Sub-Saharan Africa.

## Methods

### Data

The study made use of pooled data from most current Demographic and Health Survey (DHS) conducted from January 1, 2010, and December 31, 2016, conducted in 18 countries in Sub-Saharan Africa. DHS is a nationwide survey collected every five-year period across low and middle-income countries. DHS focuses on maternal and child health by interviewing women of reproductive age (15–49 years). DHS surveys follow the same standard procedures – sampling, questionnaires, data collection, cleaning, coding and analysis which allows for cross-country comparison. The survey employs a stratified two-stage sampling technique. The first stage involved the selecting of points or clusters (enumeration areas [EAs]). The second stage is the systematic sampling of households listed in each cluster or EA. All women in their reproductive age (15–49) who were usual of selected households or visitors who slept in the household on the night before the survey were interviewed. The response rate varied from 86.2% to 100.0% For the purpose of this, only women who had information on reproduction health decision-making were used (*N* = 84,486). Women gave oral and written consent. Ethical approval was given by individual national institutions review board and by ICF International institutional review board. Permission to use the data set was sort from MEASURE DHS. Data set is available to the public at www.measuredhs.org.

### Definition of variables

#### Outcome variables

The outcome variable employed for this study was intimate partner violence. The outcome variable was derived from three questions “experienced any sexual violence?”, “experienced any emotional violence?” and “experienced and physical violence?”. The response categories of these variables were: “Yes” and “No”. The ‘Yes’ responses were coded ‘1’ and the ‘No’ responses were coded ‘0’. An index was created with all the “Yes” and “No” answers with scores ranging from 0 to 3. The score 0 was labelled as “No” and 1 to 3 was labelled as “Yes”. A dummy variable was generated with ‘0’ score being females who had not experienced any form of sexual or emotional or physical violence and ‘1’ if females had experienced either sexual or emotional or physical violence.

#### Explanatory variables

The main explanatory variable, decision-making capacity, was derived from three questions “decision on personal health care”, “decision on large household purchase” and “decision on visits to family or relatives”. These response categories were recoded as “not alone = 0” and “alone = 1”). An index was created with all the “yes” and “no” answers with scores ranging from 0 to 3. The score 0 and 1 were labelled as “no capacity” and 2 and 3 were labelled as “capacity”. A dummy variable was generated with ‘0′ score being females who did not have the capacity and ‘1′ if females who had the capacity.

The other explanatory variables consisted of: residence, age, wealth status, education, religion, occupation, marital status, partner’s education and country. Residence was categorized as urban and rural. Age was grouped in 5 – year interval: 15–19, 20–24, 25–29, 30–34, 35–39, 40–44, 45–49. Wealth status was derived from the ownership of a variety of household assets and categorized as poorest, poorer, middle, richer and richest. Level of education and partner’s education was captured as no education, primary, secondary and higher education. Religion was recoded as Christian, Muslims and Others. Religion was not available for Niger. Occupation was categorized as not working, working outside the home and working at home. Marital status was captured as married and cohabitation.

#### Statistical analysis

Descriptive and inferential statistics were conducted. Descriptive figures are reposted in percentages by countries. Univariate and multivariate logistic regression models used to investigate the relationship between the explanatory variables and the outcome variable. Two models were used to access the predictors of intimate partner violence. Model I looked at a bivariate analysis of the main independent variable, thus, decision-making capacity and the outcome variable. Model II looked at a bivariate analysis between decision-making capacity and the outcome variable and controlled for age and country. Model III adjusted for age and country by including them in the model together with all the other independent variables. This was done to find the association between all the independent variables, including age and country and the outcome variable. All frequency distributions were weighted whiles the survey command in Stata was used to adjust for the complex sampling structure of the data in the regression analyses. All results of the logistic analyses were presented as odds ratios (ORs) with 95% confidence intervals (CIs).

## Results

### Prevalence of IPV in sub-Saharan Africa

The survey included a total of 84,486 women from 18 countries in Sub-Saharan Africa. Table 1 presents results on respondents who have ever experienced any sexual violence, any emotional violence, any physical violence and any sexual, emotional violence or physical violence.

**Table 1:** Experience of IPV among women in Sub-Saharan Africa

Country	N*	Experienced any sexual violence		Experienced any emotional violence		Experienced any physical violence		Experienced any sexual or emotional or physical violence	
		Number	Percent	Number	Percent	Number	Percent	Number	Percent
Congo DR, 2013–2014	4758	1163	24.4	1661	34.9	2127	44.7	2676	56.2
Cote d’voire, 2011–2014	4356	230	5.3	818	18.8	1087	24.9	1357	31.2
Cameroon, 2011	3442	496	14.4	1382	40.1	1491	43.3	1950	56.6
Gabon, 2012	2791	396	14.2	901	32.3	1264	45.3	1512	54.2
Gambia, 2013	3161	70	2.2	446	14.1	584	18.5	773	24.4
Kenya, 2014	3613	421	11.7	1033	28.6	1221	33.8	1569	43.4
Comoros, 2012	1979	31	1.6	138	7.0	98	4.9	186	9.4
Mali, 2012–2013	2647	356	13.4	827	31.2	779	29.4	1155	43.6
Malawi, 2010	4517	801	17.7	1181	26.1	1058	23.4	1765	39.1
Mozambique, 2011	4843	351	7.2	1591	32.8	1454	30.0	2165	44.7
Nigeria, 2013	21,413	940	4.4	3884	18.1	2860	13.4	5017	23.4
Namibia, 2015	889	56	6.4	198	22.3	190	21.3	273	30.7
Rwanda, 2014–2015	1540	152	9.8	347	22.6	434	28.2	577	37.4
Sierra Leone, 2013	4023	283	7.1	1144	28.4	1751	43.5	2011	50.0
Chad, 2014–2015	3204	298	9.3	671	21.0	773	24.1	1029	32.1
Togo, 2013–2014	4778	318	6.7	1371	28.8	896	18.8	1642	34.4
Zambia, 2013–2014	7576	1194	15.8	1648	21.8	2786	36.8	3426	45.2
Zimbabwe, 2015	4952	570	11.5	1485	30.0	1445	29.2	2172	43.9
All countries	84,486	8128	9.6	20,729	24.5	22,297	26.4	31,254	37.0

*weighted N

On the whole, the percentage of women in who had experienced any sexual violence was 9.6%, those who had experienced any emotional violence was 24.5%, those who had experienced any physical violence was 26.4% and those who had experienced any sexual, emotional violence or physical violence was 37%. Out of the 19 countries, women in Congo DR recorded the highest sexual violence (24.4%) and Comoros recorded the lowest rate of sexual violence (1.6%). A greater of percentage (40.1%) of women in Cameroon had experienced any emotional violence and the country with the lowest percentage of women who had experienced any emotional violence was Comoros (7%). With physical violence, Gabon had the highest prevalence (45.3%) while Comoros had the lowest prevalence (4.9%). Finally, 56.6% of women in Cameroon had experienced any sexual, emotional or physical violence and 9.4% of women in Comoros had experienced any sexual, emotional or physical violence (see Table 1).

### Decision-making capacity among women in sub-Saharan Africa

Three variables were used to assess women decision-making capacity. They include the decision on personal health care, the decision on a large household purchase and the decision on visits to family or relatives. In general, 12.1% of women in Sub-Saharan Africa had decision-making capacity and 87.9% had no decision-making capacity (see Table 2). Further results indicate that, out of the 18 countries in Sub-Saharan Africa, Namibia had the greatest percentage of women with decision-making capacity (32.7%), with Mali and Malawi having the least percentage of women with decision-making capacity (5.5%).

**Table 2:** Decision-making capacity among women in Sub-Saharan Africa

Country	Decision on personal healthcare		Decision on large household purchase		Decision on visits to family or relatives		*Decision-making capacity*	
	Not alone (%)	Alone (%)	Not alone (%)	Alone (%)	Not alone (%)	Alone (%)	No capacity (%)	Capacity (%)
Congo DR, 2013–2014	89.8	10.2	84.1	15.9	81.3	18.7	90.0	10.0
Cote d’voire, 2011–2014	89.9	10.1	91.1	8.9	81.5	18.5	91.2	8.9
Cameroon, 2011	84.4	15.6	83.8	16.2	77.1	22.9	86.1	13.9
Gabon, 2012	74.6	25.4	61.9	38.1	67.0	33.0	73.1	26.9
Gambia, 2013	74.6	25.4	94.6	5.5	81.7	18.3	86.0	14.0
Kenya, 2014	62.6	37.4	79.7	20.3	77.0	23.0	77.1	22.9
Comoros, 2012	81.2	18.8	75.1	24.9	72.7	27.3	79.0	21.0
Mali, 2012–2013	94.1	5.9	92.3	7.7	93.1	6.9	94.5	5.5
Malawi, 2010	81.8	18.2	90.6	9.4	82.4	17.6	94.5	5.5
Mozambique, 2011	78.9	21.1	87.2	12.8	83.3	16.7	86.0	16.0
Nigeria, 2013	93.7	6.3	94.5	5.6	92.3	7.7	94.8	5.2
Namibia, 2015	53.4	46.6	73.7	26.3	68.6	31.4	67.3	32.7
Rwanda, 2014–2015	79.3	20.7	89.5	10.6	83.7	16.3	87.3	12.7
Sierra Leone, 2013	92.5	7.5	92.8	7.2	90.8	9.2	93.7	6.3
Chad, 2014–2015	92.2	7.8	80.3	19.7	77.6	22.4	87.9	12.1
Togo, 2013–2014	88.8	11.2	85.9	14.1	83.7	16.3	90.0	10.0
Zambia, 2013–2014	68.4	21.6	88.5	11.5	79.6	20.4	85.0	15.0
Zimbabwe, 2015	65.4	34.6	72.8	27.2	73.0	27.0	74.3	25.7
All countries	83.4	16.6	87.2	12.8	83.3	16.7	87.9	12.1

### Univariate and multivariate logistic regression

As shown in Table 3, the univariate and multivariate logistic regression analyses carried out between women decision-making capacity and background characteristics and IPV showed significant associations between decision-making capacity, age, country, religion, partners’ education, occupation, marital status, education and wealth status and IPV. As shown in model I, II and III, results on women’ decision-making capacity showed that women with decision-making capacity were more likely to experience IPV compared to those who had no decision-making capacity [OR = 1.73; CI = 1.61–1.85], [OR = 1.49; CI = 1.42–1.55] and [AOR = 1.35; CI = 1.35–1.48] respectively. The odds of experiencing IPV was low among women 15–19 years compared to women in other age groups, with women aged 20–24 years having the highest odds of ever experienced IPV [AOR = 1.41; CI = 1.32–1.51]. In relation to country, women in Mali had the highest odds of ever experienced IPV compared to women in Congo DR [AOR = 1.50; CI = 1.34–1.67]. However, the likelihood of ever experienced IPV was low among women in Comoros compared to women in Congo DR [AOR = 0.17; CI = 0.15–0.21]. Religion was also found to have an influence on women experience of IPV. Specifically, women who belong to other religious groups and Christians were more likely to IPV compared to those who were Muslims [AOR = 1.73; CI = 1.65–1.82] and [AOR = 1.87; CI = 1.72–2.02] respectively. Further results also showed that women who have partners with no education [AOR = 1.11; CI = 1.03–1.20], those whose partners had primary education [AOR = 1.34; CI = 1.25–1.44] and those whose partners had secondary education [AOR = 1.22; CI = 1.15–1.30] were more likely to IPV compared to those whose partners had higher education.

**Table 3:** Logistic regression on IPV and background characteristics among women in sub-Sahara Africa

Variable	Proportions	Model I	Model II	Model III
	N = 84,486 (%)	OR (95%CI)	OR (95%CI)	AOR (95%CI)
Decision-making capacity				
No capacity	74,315 (87.9)	Ref	Ref	Ref
Capacity	10,271 (12.1)	1.73***(1.61–1.85)	1.49***(1.42–1.55)	1.35***(1.35–1.48)
Age				
15–19	6078		Ref	Ref
20–24	14,431		1.33***(1.25–1.42)	1.30***(1.22–1.39)
25–29	18,007		1.46***(1.37–1.56)	1.41***(1.32–1.51)
30–34	15,901		1.45***(1.36–1.55)	1.39***(1.30–1.49)
35–39	13,095		1.42***(1.32–1.52)	1.35***(1.25–1.44)
40–44	9484		1.34***(1.24–1.44)	1.26***(1.17–1.36)
45–49	7490		1.24***(1.15–1.34)	1.17***(1.09–1.28)
Country				
Congo DR	4758		Ref	Ref
Cote d’voire	4356		0.36***(0.33–0.39)	0.49***(0.45–0.54)
Cameroon	3442		1.03(0.95–1.13)	1.21***(1.10–1.32)
Gabon	2791		0.91**(0.83–1.00)	0.93(0.85–1.03)
Gambia	3161		0.30***(0.27–0.33)	0.64***(0.58–0.71)
Kenya	3613		0.53***(0.48–0.58)	0.61***(0.56–0.66)
Comoros	1979		0.08***(0.07–0.09)	0.17***(0.15–0.21)
Mali	2647		0.65***(0.59–0.71)	1.50***(1.34–1.67)
Malawi	4517		0.50***(0.46–0.55)	0.55***(0.51–0.60)
Mozambique	4843		0.66***(0.60–0.71)	0.77***(0.71–0.84)
Nigeria	21,413		0.27***(0.25–0.28)	0.41***(0.39–0.44)
Namibia	889		0.33***(0.29–0.38)	0.45***(0.39–0.52)
Rwanda	1540		0.45***(0.40–0.51)	0.42***(0.37–0.47)
Sierra Leone	4023		0.74***(0.68–0.81)	1.39***(1.27–1.53)
Chad	3204		0.30***(0.28–0.33)	0.57***(0.51–0.63)
Togo	4778		0.45***(0.41–0.49)	0.50***(0.46–0.55)
Zambia	7576		0.66***(0.62–0.71)	0.70***(0.65–0.76)
Zimbabwe	4952		0.56***(0.51–0.60)	0.66***(0.60–0.71)
*Residence*				
Urban	30,654 (36.3)			Ref
Rural	53,832 (63.7)			0.88 (0.85–0.92)
*Religion*				
Islam	31,697 (37.5)			Ref
Christian	49,068 (58.1)			1.73***(1.65–1.82)
Others	3721 (4.4)			1.87***(1.72–2.02)
*Partner’s Education*				
No education	25,090 (29.7)			1.11**(1.03–1.20)
Primary	23,164 (27.4)			1.34***(1.25–1.44)
Secondary	38,159 (33.3)			1.22**(1.15–1.30)
Higher	8073 (9.6)			Ref
*Occupation*				
Unemployed	27,164 (32.1)			Ref
Employed	57,322 (67.9)			1.33***(1.28–1.37)
*Marital status*				
Married	73,338 (86.8)			Ref
Cohabitation	11,148 (13.2)			1.16***(1.10–1.21)
*Education*				
No education	31,043 (36.7)			1.37***(1.24–1.51)
Primary	27,441 (32.5)			1.65***(1.50–1.82)
Secondary	21,983 (26.0)			1.50***(1.37–1.64)
Higher	4019 (4.8)			Ref
*Wealth status*				
Poorest	17,075 (20.2)			1.28**(1.20–1.37)
Poorer	17,271 (20.5)			1.24**(1.17–1.32)
Middle	16,552 (19.6)			1.27***(1.20–1.34)
Richer	16,904 (20.0)			1.11**(1.06–1.17)
Richest	16,684 (19.7)			Ref

*p < 0.10 **p < 0.05 ***p < 0.001 *OR* Odds Ratio

The odds of experiencing IPV were high among women who were employed compared to those who were unemployed [AOR = 1.33; CI = 1.28–1.37]. The likelihood of the occurrence of IPV was also high among women who were cohabiting compared to those who were married [AOR = 1.16; CI = 1.10–1.21]. Women with no education [AOR = 1.37; CI = 1.24–1.51], those with primary education [AOR = 1.65; CI = 1.50–1.82] and those with secondary education [AOR = 1.50; CI = 1.37–1.64] were more likely to experience IPV compared to those with higher education. Finally, women with poorest wealth status [AOR = 1.28; CI = 1.20–1.37], those with poorer wealth status [AOR = 1.24; CI = 1.17–1.32], those with middle wealth status [AOR = 1.27; CI = 1.20–1.34] and those with richer wealth status [AOR = 1.11; CI = 1.06–1.17] were more likely to IPV compared to women with richest wealth status.

## Discussion

In this present study, our aim was to examine the association between women decision-making capacity and IPV among Women in Sub-Saharan Africa. We also looked at how socio-demographic factors influence IPV among Women in Sub-Saharan Africa. Our study found an overall prevalence of IPV of 37% and this is consistent with findings of previous studies on the prevalence of IPV in Sub-Saharan region [1, 25, 31]. These previous however included the general regions in Africa but our study considered only women in Sub-Saharan Africa. The comparison of the study results is, therefore, to be made with caution.

On women decision-making capacity, the current study used three variables (the decision on personal health care, the decision on a large household purchase and the decision on visits to family or relatives) and this was linked to women empowerment. It was revealed that the empowerment level of women in Sub-Saharan Africa is low (12.1%). The results further revealed that out of the 19 countries in Sub-Saharan Africa, Namibian women were those who were more empowered (32.7%) while Mali and Malawi had the least percentage of empowered women (5.5%). The finding of our study in relation to the level of women empowerment mirrors the findings of Ameyaw et al. [32]. The differences in the level of women empowerment could also be due to the variations in policies and legislation that exist in countries in Sub-Saharan Africa.

Our data suggest that women who had the capacity to take decisions were more likely to experience IPV compared to those who did not have decision-making capacity. The positive association between women decision-making capacity and IPV is consistent with a current study by Cools and Kotsadam [4]. The finding still corroborates previous studies that found that the more a woman is empowered the more likely she is to suffer from IPV [25–27, 30]. The possible explanation for this is the fact that women who are more empowered are able to fight for their rights and will not accept men to fully dictate to them which could result in IPV. Another possible explanation is that in Africa, most cultures prefer women to be subordinated to men, but women who are more empowered do not solely depend on men for their survival and tend to resist some of the decisions of men which may bring about intimate partner violence. However, the findings are contrary to the findings of a Bangladeshi study where women empowerment is served as protective means to get rid of IPV [29].

The study revealed that women aged 15–19 years are less likely to report having ever experienced IPV compared with older women. Similar findings were obtained by Bazargan-Hejazi et al. [33] who found that younger women were less likely to report IPV compared to women in the ages 45 to 49. Similarly, a study on women empowerment and spousal violence in relation to health outcomes in Nepal also identified that youngest women (15–19) are the least likely to report having ever experienced IPV compared with older women [34]. A likely explanation is that young women duration of exposure to the risk of spousal violence is less than that of older women. Hence, the likelihood of experiencing IPV is low among them. Furthermore, women in the older age groups tend to be less educated, have more children, and are more likely to have lower decision-making capacity than their slightly younger counterparts, hence are more likely to have higher levels of IPV. Older women are also more likely to have experienced spousal violence during their lifetime, compared to those aged 15–19 whose lifetime prevalence of IPV is likely to be lower.

Another key finding was the influence of religious affiliation on women experience of IPV in Sub-Saharan Africa. A number of studies have shown the association between religious affiliation and intimate partner violence [35, 36]. In most cases, religious affiliation and groups can be social networks that involve the sharing of ideas and behaviours. It was shown from our study that, women who belonged to other religious groups and Christians were more likely to experience IPV compared to those who were Muslims. The findings are consistent with other studies such as Naved and Persson [35] and Ackerson et al. [36]. Christianity and other religious groups are sometimes associated with dogmatic views regarding gender inequality, the centrality of male dominance in various spheres of life in the home, and also the sanctity of family unity, that have the potential to be misinterpreted or exaggerated. The misinterpretation of these dogmatic views may bring about IPV against women. Furthermore, another reason for this finding could be that the Islamic religion has strict norms that may prohibit violence against women. Such norms relate to how women should be treated with diligence within the religion.

The study revealed that there was an inverse association between the educational level of a woman’s partner and IPV. The odds of being abused decreased with an increasing level of women partners’ level of education. Women who have partners with no formal education, those whose partners had primary education and those whose partners had secondary education were more likely to experience IPV compared to those whose partners had higher education. The possible explanation to this is that those whose partners have higher education are exposed to training and they value the need to respect the rights and freedom of other people. Again, the education that they receive makes them get rid of certain socio-cultural practices that put limitations on the value of women. The relationship between spousal educational level and IPV is not consistent in literature. Our findings are consistent with the findings of studies that showed that as a partner’s level of education increases the risk of experiences sexual or emotional violence decreases [28, 37, 38]. Nonetheless, the findings do not corroborate the findings of Burazeri et al. [39]. In some instances, some men who are less educated might have low self-esteem and this may lead to the tendency of being violent against their partners [28].

The odds of experiencing IPV were high among women who were employed compared to those who were unemployed. This finding can be explained by the fact that if women attain a higher level of formal education, they are also empowered economically. As a result, they can compete with men for available positions and jobs that are available in society and this does not make them rely on men for their basic needs in life. Again, educational level of a woman can predict the type of occupation she will take up. Because occupation is one of the determinants of empowerment, women who are employed tend to resist being controlled by their men. Also, the study found that women with no education, those with primary education and those with secondary education were more likely to experience IPV compared to those with higher education. It is a well-established fact that if women are more educated, due to the exposure they obtain, it can let them fight for their rights and that of other marginalized women in their communities. They can help champion the fight against indiscrimination and bad socio-cultural practices that are against women. The findings of this current study are congruent to that of Hindin, Kishor and Ansara [40], who found education as a protective factor against sexual or emotional violence in Bolivia, Kenya and Zimbabwe. It can, therefore, be assumed that encouraging more women to be educated can reduce the prevalence of IPV.

Furthermore, in our study, there was a significant association between marital status and IPV. The results showed that the likelihood of the occurrence of IPV was high among women who were cohabiting compared to those who were married. This could be clarified in the sense that those in marriage usually understand each other and are more likely to compromise on certain issues which brings less conflict in their matrimonial homes. The findings are not consistent with the findings of that currently married women are the most likely to report physical or sexual violence in the last 12 months [41].

In our analysis, wealth status showed a statistically significant influence on IPV. Specifically, women with poorest wealth status, those with poorer wealth status, those with middle wealth status and those with richer wealth status were more likely to experience IPV compared to women with richest wealth status. This means that IPV decreases when there is an increase in wealth status. The relationship between wealth status and IPV is however not clear. Some studies have found a positive relationship while others have found an inverse relationship. The findings of this present study are similar to other studies conducted in Sub-Saharan Africa [42]. However, the findings of this current study deviated from that of Bamiwuye and Odimegwu [43] in their study in Zambia and Mozambique and Okemgbo, Omideyi and Odimegwu [44] in their study in Nigeria.

The study had its strength from the large data used from different countries in Sub-Saharan Africa. The use of nationally representative surveys (DHS) and the use of stratified two- stage sampling technique made it possible to obtain samples that are highly representative of the target populations. The use of a large sample size and the national representative nature of the data make conclusions from our study valid. Nonetheless, the study sample was limited to only women in their reproductive age group (15-49 years old) and did not consider women below 15 years and those above 49 years. Again, the cross-sectional nature of the study does not make it possible to make any causal inference but rather only associations can be made. The data also relies mainly on a verbal report that was given by the women. Another limitation of the study is the use of lifetime prevalence instead of prevalence within the last 12 months. This affected the odds of IPV among respondents, especially with respect to age. Finally, since data was obtained using a purely quantitative approach, in-depth information which could have been obtained using a qualitative approach was not considered.

## Conclusion

African women are burdened with IPV in various stages of their life course, especially during their reproductive ages. Our study has examined the association between women decision-making capacity and IPV among Women in Sub-Saharan Africa. It was found that the prevalence of IPV is Sub-Saharan Africa is within the range of prevalence found in previous studies that have been conducted in Africa. In Sub-Saharan Africa, women in Cameroun experienced the highest lifetime prevalence of IPV. Our study also showed a very low level of women decision-making capacity in Sub-Saharan Africa. The country with the high level of decision-making capacity in Sub-Saharan Africa even had a percentage of decision-making capacity far below 50% of the women in their reproductive age group. Though related socio-demographic characteristics and women decision making capacity provided an explanation of IPV among women in Sub-Saharan Africa, there were differences in relation to how each socio-demographic variable predisposed women to IPV in Sub-Saharan Africa.

The odds of women experiencing IPV was higher among women who belonged to other religious groups, whose partners had primary education, women who were employed, women who were cohabiting, women with primary education and those with middle wealth. The findings thus clearly indicate the need for governments in Africa to invest more in female child education since an inverse relationship between education and IPV was identified. There is also the need for timely screening of women for physical violence but also since that was the most prevalent type of violence women experienced in Sub-Saharan Africa. Although little evidence shows that screening alone has the propensity to reduce the prevalence of IPV, when this is coupled with education of males on the effects of IPV, it is more likely to reduce the occurrence of IPV in future. The risk of IPV among women who are empowered and those who are employed can be reduced by sensitizing women to understand the need to be submissive no matter their status in society. Chastisement that comes with offenders of IPV should be carried out and enforced so as to deter other people from engaging in such acts.
